# Proxy indicators for antibiotic consumption; surveillance needed to control antimicrobial resistance

**DOI:** 10.2471/BLT.18.227348

**Published:** 2019-01-01

**Authors:** Wenjing Tao, Verica Ivanovska, Birgitta Schweickert, Arno Muller

**Affiliations:** a Essential Medicines and Health Products, World Health Organization, avenue Appia 20, 1211 Geneva 27, Switzerland.

The rapid spread of antibiotic resistance is a major public health challenge, because it endangers our ability to treat potentially life-threatening infections. In response to this challenge, in 2015 the World Health Assembly adopted the *Global action plan on antimicrobial resistance* to ensure sustainable access to effective and safe antimicrobial medicines.^1^

This action plan recognizes surveillance of antibiotic consumption and antibiotic resistance in humans and animals as a key strategy in addressing antibiotic resistance. Surveillance of antibiotic consumption can help countries identify problems relating to antibiotic use; direct their interventions to tackle these problems; and evaluate these interventions. Over time, surveillance allows comparisons between and within countries that help set common targets and identify changing trends in antibiotic use patterns.

Success stories where surveillance data have contributed to the improvement of antibiotic use are well documented. For example, surveillance data helped the governments of Belgium and France to realize that antibiotic consumption in their respective countries was among the highest in Europe, prompting them to launch national awareness campaigns that proved effective in reducing antibiotic prescribing.^2^ Australia and Slovenia applied restrictions on the prescription of specific antibiotics and used surveillance data to assess the impact of such restrictions.^3^ To discourage perverse financial incentives, the Republic of Korea removed the right of the prescribing doctor to also dispense antibiotics; national surveillance data suggests that such policy resulted in better prescribing practices and less antibiotic use.^3^ Access to surveillance data on antibiotic consumption and antibiotic resistance in humans and animals has helped countries to better understand the relationship between human and animal antibiotic consumption and its impact on the development of antibiotic resistance.^4^^,^^5^

While many high-income countries have ongoing surveillance of national antibiotic consumption, data from most low- and middle-income countries is absent. The lack of data on the quantity of antibiotics consumed at the national level is concerning: inappropriate prescribing and self-medication with antibiotics are prevalent in many of these countries,^6^ leading to high rates of antibiotic resistance.^7^ To address this gap, the World Health Organization (WHO) developed a standardized surveillance protocol for countries without existing national surveillance of antibiotic consumption, using aggregated sales data. WHO also supported these countries in implementing this protocol.^8^ National data submitted to WHO was collated in the first *WHO Global report on antibiotic consumption*, released in November 2018, during the World Antibiotic Awareness Week.^9^

The report presents 2015 data on the consumption rates of antibiotics in 65 countries and areas, including 15 low- and middle-income countries that collected such data in a systematic manner for the first time with support from WHO.^9^ Among the key findings is a 16-fold difference in antibiotic consumption among the countries included in the report, confirming the significant variations observed in a previous study using national sample surveys of antibiotic sales.^10^ The report presents data according to the WHO Model List of Essential Medicines’ categorization of antibiotics, which classifies antibiotics into three groups – access, watch and reserve – based on their treatment profile and potential for causing resistance.^11^ Access antibiotics, which represent first- and second-line therapy for common infections, were the most consumed antibiotics in most countries. Watch antibiotics, with higher potential for causing antibiotic resistance, accounted for 20% to 50% of national antibiotic consumption. Consumption of reserve antibiotics, which should be kept for last-resort treatment of severe infections, were almost exclusively reported in high-income countries.^9^ The observed variations between countries indicate that there is insufficient access to these life-saving medicines in some countries, and overuse of the same medicines in others.

The recent implementation of surveillance on antibiotic consumption in the 15 low- and middle-income countries has also had a positive effect on strengthening pharmaceutical systems. For example, Côte d’Ivoire introduced a system to assign codes to authorized medical products to improve medicines tracking. Bangladesh is planning to improve quality assurance of medicines by prioritizing controls of the most sold antibiotic medicines identified in its surveillance data.^9^

While such progress is encouraging, there is a need for continued global commitment to support countries in developing national surveillance of antibiotic consumption across all sectors, including livestock and agriculture. Countries that already have existing surveillance systems should continue to share their data with WHO and the World Organisation for Animal Health (OIE), which oversees surveillance of antibiotic consumption by animals.^12^ Data on antibiotic consumption across all sectors can support effective national and global coordination of actions to curb antibiotic resistance and help prioritize areas where these actions are most critical.
